# Historical Typhoon Search Engine Based on Track Similarity

**DOI:** 10.3390/ijerph16244879

**Published:** 2019-12-04

**Authors:** Meng-Han Tsai, Hao-Yung Chan, Chun-Mo Hsieh, Cheng-Yu Ho, Hung-Kai Kung, Yun-Cheng Tsai, I-Cheng Cho

**Affiliations:** 1 Department of Civil and Construction Engineering, National Taiwan University of Science and Technology, Taipei City 10607, Taiwan; d10705005@mail.ntust.edu.tw; 2 Department of Economics, National Taiwan University, Taipei City 10617, Taiwan; b06303059@ntu.edu.tw; 3 Department of Geography, National Taiwan University, Taipei City 10617, Taiwan; b06208030@ntu.edu.tw (C.-Y.H.); b06208001@ntu.edu.tw (H.-K.K.); 4 School of Big Data Management, Waishuanghsi Campus, Soochow University, Taipei City 11102, Taiwan; pecutsai@scu.edu.tw; 5 Department of Civil Engineering, National Taiwan University, Taipei City 10617, Taiwan; uscedula@gmail.com

**Keywords:** decision support, typhoon track similarity, search engine, user interface design

## Abstract

The potential effect of a typhoon track on the extent of damage makes the track a critical factor during the emergency response phase. Historical typhoon data may provide information for decision makers to anticipate the impact of an upcoming typhoon and develop prevention strategies to reduce the damage. In our preliminary work, we proposed a track similarity algorithm and implemented a real-time search engine for past typhoon events. However, the proposed algorithm was not discussed thoroughly in the preliminary work, and the great number of historical typhoon track records slowed down the similarity calculations. In addition, the tool did not feature the option of automatically importing upcoming typhoon track predictions. This research introduces the assumption of the recentness dominance principle (RDP), explores the details of the track similarity algorithm of the preliminary work, completes the discussion of parameter setting, and developed a method to improve the efficiency of the similarity calculation. In this research, we implemented the proposed advanced methodology by developing a new information display panel featuring the ability to auto-import forecast data. The results of this study provide decision makers and the public with a concise and handy search engine for searching similar historical typhoon records.

## 1. Introduction

The interaction of a typhoon and the land affects the winds and precipitation of different areas, causing different amounts of damage. The potential effect of a typhoon track on the extent of damage makes the track a critical factor during the emergency response phase. Historical typhoon data, covering tracks, speeds, and precipitation distributions, may provide information for decision makers to anticipate the impact of an upcoming typhoon and develop prevention strategies to reduce the damage caused by the typhoon. By categorizing historical typhoon tracks, the interactions of the typhoons with the landforms and the patterns of damage can be analyzed. The Central Weather Bureau (CWB) of Taiwan has analyzed historical records and determined nine categories of tracks of typhoons that have hit Taiwan, covering approximately 96% of historical typhoons that affected Taiwan between 1911 and 2018 [1]. Moreover, the CWB has linked the behavior of wind and precipitation of typhoons with different tracks to different regions in Taiwan [2,3]. The patterns found may help decision makers predict the damage that will be induced by upcoming typhoons.

In our preliminary work, we proposed a track similarity algorithm and implemented a real-time search engine for past typhoon events. A cross-platform user interface was designed and validated by a usability test, and a three-year field test was coordinated with the government of Taiwan. The search engine helped the relevant personnel to intuitively search and review the historical typhoon information and efficiently develop corresponding response strategies. However, the proposed algorithm was not discussed, leaving the choice of parameter settings baseless. In addition, the large number of historical typhoon track records resulted in long calculation times for the track similarity algorithm. Further, the implemented tool did not have a feature for automatically importing the data of upcoming typhoon track predictions, forcing users to input the track manually, and reducing the convenience of the tool.

This research aimed to explore the details of the track similarity algorithm of our preliminary work, complete the discussion on parameter setting, and develop a method to improve the efficiency of the track similarity calculation. Additionally, a new information display panel was developed featuring auto-importing forecast data. A literature review of typhoon track categories and databases is presented in Section 2, including a discussion of our preliminary work. The details of the algorithm are discussed in Section 3. The implementation of the new panel and the method used to increase the efficiency of the algorithm are described in Section 4. The parameter setting is discussed in Section 5.

## 2. Literature Review

Reviewing past typhoon records is now one of the main approaches for typhoon status prediction [4]. Wu and Kuo [5] stated that the interaction of the typhoon circulation and the complicated topography related to the Central Mountain Range in Taiwan produces significant mesoscale variations in pressure, wind, and precipitation distributions in Taiwan. Huang et al. [6] investigated the link between typhoon tracks, precipitation patterns, and flood peak time, finding that different typhoon tracks appear to have preferable precipitation types. For the emergency response phase of typhoon events in Taiwan, decision makers and responders seek the status of the disaster, the response strategies, and other relevant data of similar past typhoons.

Studies on typhoons have focused more on the accuracy of forecast models in regard to different aspects. Classical methods of predicting a typhoon’s behavior are climatological or dynamic with different considerations. For example, Lee et al. [7] developed a climatology model for predicting typhoon precipitation, which provides hourly rainfall at any station or any river basin for a given typhoon center. Recently, some studies have attempted to adapt data-driven methods to forecast typhoons. For example, Rüttgers et al. [8] adapted a generative adversarial network (GAN) with satellite images as inputs for typhoon track predictions, reducing the errors between predicted and real typhoon centers measured quantitatively to kilometers. Precise forecasts may support decision-making. However, the connections between the anticipated damage from upcoming typhoons and forecasts are often implicit. Forecasts may provide the predicted amount of precipitation and the intensity of the wind, but deducing predicted damage from the prediction of precipitation and wind requires the decision makers’ knowledge of the regional capacity. In addition, the computation time for forecasting may hinder immediate reactions to emergencies. Thus, the use of past typhoon records for developing quicker response strategies may be useful for decision makers.

Currently, several typhoon databases have been developed, providing comprehensive information about past typhoon records. For tropical cyclones (TCs) in the western North Pacific (WNP), CWB developed the Taiwan Typhoon Database, containing observational data of past typhoon events during 1958–2019 [9]. In addition, the National Institute of Informatics (NII) of Japan manages the Digital Typhoon Database, which has archives of the data on past typhoons in the WNP during 1951–2018 and the western South Pacific during 1907–2018 [10]. The databases provide several search methods, including by time, pressure, wind, location, rainfall, intensity, etc. However, such databases do not provide the ability to search by typhoon track. Tsai et al. [4] remarked that efficient search methods should allow users to search past typhoon records by comparing the tracks between upcoming and past typhoons. The track of a typhoon is a critical factor since it may affect the extent of the damage.

Alternatively, some studies have categorized typhoons by clustering tracks. Camargo et al. [11] applied probabilistic clustering analysis to categorize TCs in the WNP during 1950–2002 into seven distinct types. Chu et al. [12] proposed a track-pattern-oriented categorization approach to forecasting TC frequency in the WNP, clustering the historical TC tracks into seven distinct types. Kim et al. [13] applied a fuzzy c-means method to cluster TC tracks and obtained seven clusters using TC records in the WNP during 1965–2006. Chu et al. [14] integrated fuzzy cluster analysis and kernel density estimation to cluster the typhoon tracks using typhoons that affected Taiwan during 1986–2010, finding six clusters. Ho et al. [15] targeted seasonal TC track clusters covering the entire WNP and found seven clusters. Kim et al. [16] developed a statistical–dynamical model for TC intensity prediction using a track-pattern clustering (TPC) method and ocean-coupled potential predictors, finding five clusters using TC tracks during 2004–2012 in the WNP. The CWB categorized the typhoon tracks into nine specific categories and one special category using typhoon data from the past 100 years [1]. Such approaches to categorizing typhoon tracks provide the possibility of accessing the databases by track categories. For example, the Taiwan Typhoon Database has a search features that allows searching by the ten categories of typhoon tracks proposed by the CWB. However, even though many studies have been conducted to cluster typhoon tracks, a users’ determination of which category a forecast track should belong to is still required. The lack of integration with forecast data and historical typhoon databases makes the task of track matching less intuitive for users.

T-search, a real-time historical typhoon search engine, was developed by the authors to assist the disaster management staff in predicting upcoming damage and developing strategies for emergency response [4]. The sorting mechanism is based on a track similarity algorithm also developed in the preliminary work. An intuitive user interface was designed for the search engine to increase the efficiency of reviewing the historical typhoon records. The capability of T-search for aiding the staff in accomplishing their tasks was validated by a usability test involving nine subjects. Moreover, a three-year field test was conducted in cooperation with the government of Taiwan for a real case study, demonstrating three major advantages of T-search for the typhoon emergency response activities: the enhanced accessibility to typhoon records, the improved efficiency of utilizing historical information, and the opportunity for decision makers to develop multiple strategies. However, the role of each parameter of the algorithm was not discussed throughout, leaving the choice of parameter setting baseless. Additionally, there are approximately 1700 typhoon records whose tracks are composed of 65,000 coordinates on average, slowing down the similarity calculation. In addition, the tool did not have a feature for automatically importing the data of upcoming typhoon track predictions, forcing users to input the track manually, thus lowering the convenience of using the tool.

## 3. Methodology

This research extracted the core of the model in the preliminary work with the presumption of the recentness dominance principle (RDP). The RDP states that those later user-specific track points are more relevant than the earlier ones, and thus their relative weighting should be increased. The RDP is constructed on the presumption that when the typhoon is in its developing stage, the typhoon’s direction possesses high uncertainty. This research elaborates on the preliminary work and provides the definition and interpretation of the time weighting. We discuss the core of the comparison model by decomposing it into a static sector and a dynamic sector, propose a strategy to enhance the efficiency of the computation, and set an order to ensure the discrimination of similarity.

### 3.1. The Core of the Comparison Model

The model retrieves the most similar historical typhoon tracks for the user-specific track of the forecasted typhoon through track comparison and matching. For each past typhoon, the model calculates the similarity based on points of tracks, valid regions given by the user, time, etc., for the user-specific track points and quantifies scores for comparison. The similarity of the forecasted typhoon and each historical typhoon is calculated with Equations (1) and (2):

(1) $$\begin{array}{cc} similarity(i) & =\sum_{k=1}^{M}\left(1+kw\right)\times \delta_{k}\\ \; & =\sum_{k=1}^{M}\delta_{k}+w\sum_{k=1}^{M}\left(k\times \delta_{k}\right) \end{array}$$

(2) $$\delta_{k}=\left\{\begin{array}{cc} 1, & d_{jk}<R_{k}\\ 0, & \mathrm{otherwise}. \end{array}\right.$$

*M* is the number of the user-specific track points. *k* is the index of the user-specific track points starting from one; for example, the initial point is given as one, while the second point is given as two, and so on. *i* is the index of the historical typhoons. *j* is the index of the closest track point of the historical typhoon *i* to the user-specific track point *k*. *w* is the time weighting of all the user-specific points, which is critical in determining the comparison result and changes depending on the value of *M*. Further details on *k* and *w* are given in Section 3.2. The binary variable $\delta_{k}$ represents whether the historical typhoon track point *j* falls in the given valid region of the user-specific track point *k*. The valid regions corresponding to the user-specific track points are designed to restrict the distances between a forecast track point and historical track points (denoted as $d_{jk}$ in Equation (2)), and eliminate those whose tracks are too far from the user-specific track points. For each historical typhoon, at least one point should lie within the valid region set by the user-specified radius (denoted as $R_{k}$ in Equation (2)), meaning that the typhoon is close enough to the user-specific track. For the historical typhoons that do not have any track points located in the given valid regions, the similarity scores of the typhoons are zero since they are determined to be too far.

**Figure 1:**
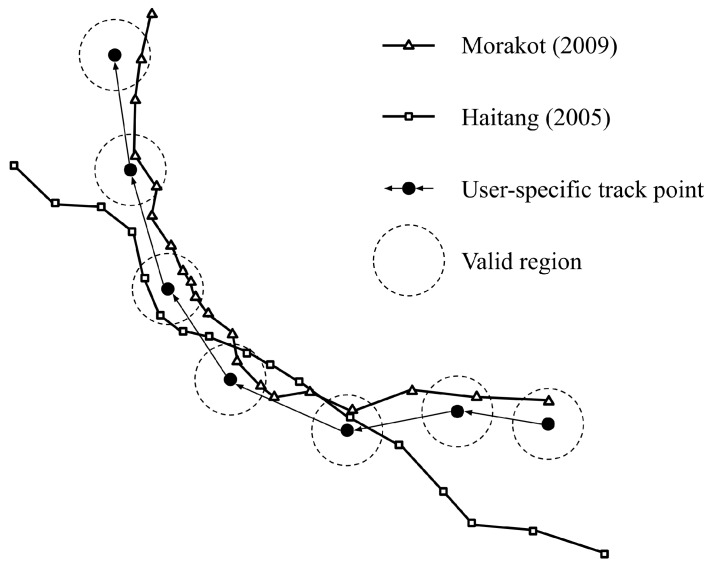
An example of the tracks of Typhoon Morakot (2009) and Typhoon Haitang (2005).

The model is capable of quantifying the similarity score of each historical typhoon, which yields a ranking and eventually outputs the most similar historical typhoon(s). For example, the scores of Typhoon Morakot (2009) and Typhoon Haitang (2005) can be simplified as 7+28w and 3+12w, as demonstrated in Figure 1. Typhoon Morakot is much more similar to the track in question than Typhoon Haitang, and thus Typhoon Morakot has a higher rank.

**Figure 2:**
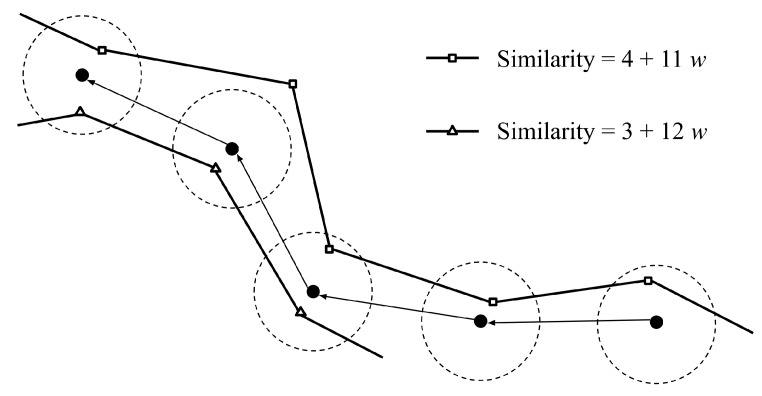
An example of two typhoon tracks for the similarity comparison demonstrating increased complexity over the example in Figure 1.

However, not every situation is as simple. A second example is shown in Figure 2, with one typhoon passing through the last three input points with the score of 3+12w, and the other passes through the first three input points and the last input point with the score of 4+11w. In the case, we cannot determine which typhoon ranks higher if the variable *w*, the time weighting, remains unknown. If the variable *w* is greater than 1, the typhoon with the score of 3+12w obtains higher similarity; by contrast, the typhoon with the score of 4+11w obtains higher similarity if the variable *w* is less than 1. Therefore, it is essential to dig deeper into the above summation for a comprehensive explanation.

### 3.2. Static Sector and Dynamic Sector

To examine the roles of *k* and *w*, we divided Equation (1) into two sectors: the static sector and the dynamic sector. The static sector refers to the constant part ($\sum_{k=1}^{M}\delta_{k}$), and the dynamic sector refers to the second component ($w\sum_{k=1}^{M}k\times \delta_{k}$).

The static sector is obtained from the number of eligible track points that are located in the given valid regions. In this sector, only the valid regions of the given track points are of concern, which present a clear view of the coarse similarity of that historical typhoon and the given track. Following the example of Typhoon Morakot, the score of 7+28w of Typhoon Morakot indicates that it passes seven given track points in total. Hence, the static sector self-explains the stationary aspect of the similarity.

The dynamic sector includes time as a factor into the model through multiplying the time series (*k*) by the time weighting (*w*). In this approach, by adjusting the given time weighting, the model emphasizes the importance of time by adding the dynamic sector to the similarity comparison. Following RDP, the value of the time weighting should be changed when more track points are specified. If the time weighting does not correlate with the number of given track points, the scores of the historical typhoons can be distorted in some cases.

To prevent the distortion, the global recentness dominance time weighting (gRDW) and the individual recentness dominance time weighting (iRDW) are introduced. They are obtained by the following approach (Equations (3) and (4)):

(3) $$\hat{w}=\max\{w_{i}:i=1..|P|\}$$

(4) $$w_{i}=\frac{s_{i,b}-s_{i,a}}{d_{i,a}-d_{i,b}}\forall \left(s_{i,a}+d_{i,a}w,s_{i,b}+d_{i,b}w\right)\in P$$

(5) $$indeterministic\;score\;pair\equiv \left(s_{a}+d_{a}w,s_{b}+d_{b}w\right)\forall a,b\in N\left(s_{a}>s_{b}\wedge d_{a}<d_{b}\wedge a<b\right).$$

For those which do not belong to the indeterministic score pairs (denoted as P in Equations (3) and (4)), which are defined in Equation (5), any values of gRDW (denoted as $\hat{w}$ in Equation (3)) greater than zero are suitable for the comparison. By contrast, when examining the indeterministic score pairs, gRDW should be carefully measured. For simplicity, the model equalizes the score of each score pair to obtain iRDW (denoted as $w_{i}$ in Equations (3) and (4)). Afterward, in an attempt to emphasize the recentness, the model takes the maximum of the set of iRDW as gRDW. After determining gRDW, there exists no indeterministic score pair that contravenes RDP, and the similarity scores can then be calculated. In Section 5, this research relaxes the assumption of RDP and presents an insight into the role of time weighting.

Taking five user-specific points (M=5) for instance, all valid combinations of static and dynamic sectors of any historical typhoon are shown in Table 1. If the time weighting (*w*) remains unknown, some indeterministic scores exist, causing the comparison result to be undetermined. In this case, there are twelve indeterministic score pairs in total, as listed in Table 2. Using Equation (4), the set of iRDW is $\left\{1,\frac{1}{2},1,1,\frac{1}{2},\frac{1}{3},1,\frac{1}{2},1,1,\frac{1}{2},1\right\}$, as listed in Table 2; therefore, the gRDW is 1.

### 3.3. Computation Optimization

The task of comparing all the indeterministic score pairs one by one is inefficient, since it requires too many computational resources and too much time. The more user-specific track points that are given, the more indeterministic score pairs there are.

Using Equation (4), we derived that for any indeterministic score pair ($s_{a}+d_{a}w$) and ($s_{b}+d_{b}w$) where $s_{a}<s_{b}$ and $d_{a}>d_{b}$, gRDW is the maximum value of all $s_{b}-s_{a}$ when $d_{a}-d_{b}$ equals 1. To enhance the efficiency, the model first compares the indeterministic score pairs for which the static sectors are 1 ($C_{1}^{M}$) to M−1 ($C_{M-1}^{M}$). If gRDW equals $s_{a}$, the model takes the maximum of the dynamic sectors $d_{a}$ of $C_{s_{a}}^{M}$, compares it with the combination of all values of the static sectors larger than $s_{a}$, and finds the maximum of the static sectors $s_{b}$ so that its dynamic sector may be not greater than $d_{a}$. Thus, the model is capable of minimizing the time by comparing column by column. By this means, the difference between the values of these two static sectors, $s_{b}-s_{a}$ is the maximum of all $w_{i}$ for which the total passing point is $s_{a}$, expressed by $\hat{w}$. By conducting the procedure column by column, the corresponding $\hat{w}$ is the maximum of all $w_{i}$. In short, this approach simplifies the process from comparing $w_{i}$ of entire indeterministic score pairs to comparing the scores column by column based on their individual greatest values of the dynamic sector. Subsequently, the model substitutes the $\hat{w}$ into the dynamic sectors and calculates the final similarity scores. Finally, the model singles out the requested results from the top following the comparison order discussed in Section 3.4.

**Figure 3:**
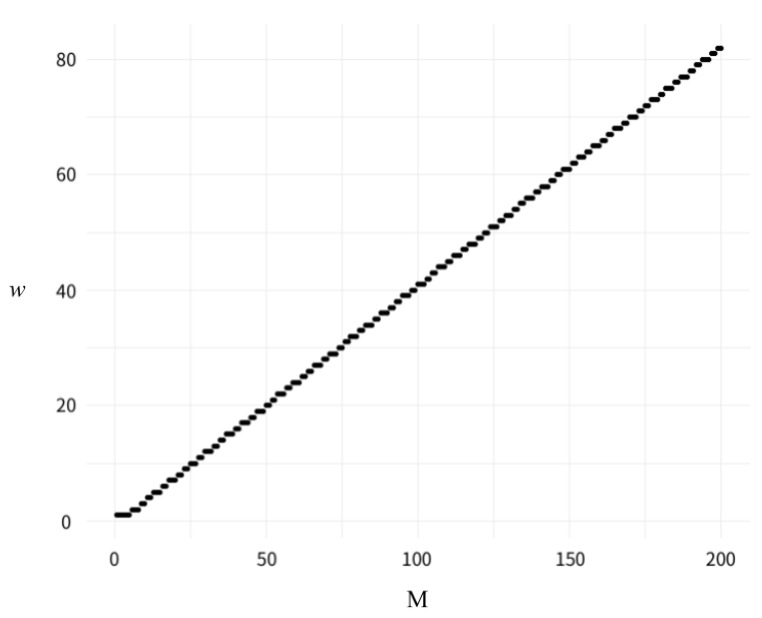
Visualization of the relationship between *M* and global recentness dominance time weighting (gRDW).

Taking M=6 for instance, the possible scores of a historical track are shown in Table 3. When the value of the static sector is 1 ($s_{a}=1$), the maximum of $d_{a}$ is 6. Then, the model goes to the next column to compare with the next value of the static sector. $d_{b}=5$ is found to satisfy the condition of $d_{a}-d_{b}=1$ when the value of the static sector is 2 ($s_{b}=2$). However, when the value of the static sector is greater than 2, there is no $d_{b}$ less than $d_{a}$, leading to an indeterministic score pair. Hence, $w_{i}$ is 1 when M=6 and $s_{a}=1$. By contrast, when the value of the static sector is 2 ($s_{a}=2$), the largest $d_{a}$ is 11. Thus, the $\hat{w}$ is 2 as $d_{b}$ is 10 when $s_{b}$ equals 4. After comparing column by column, from $s_{a}=1$ to $s_{a}=M-1=5$, the model gathers a set of iRDWs, and the maximum value of the set stands for the gRDW. In this case, when M=6, $\hat{w}$ is 2. The results are presented in Figure 3, with *M* on the x-axis and gRDW on the y-axis, which resembles the form of a Gaussian function in which gRDW is an increasing integer with increasing *M*.

### 3.4. Comparison Order

To ensure the discrimination of similarity between the historical typhoon tracks and the user-specific track, the comparison order is in five steps as follows:

1. Total similarity score;
2. Dynamic sector;
3. Static sector;
4. Month disparity;
5. Year gap.

The model first sorts the past typhoons by the computed similarity scores with the user-specific track using Equation (1). The similarity score takes both the numbers of track points of the given track the historical typhoon passes through and RDP into consideration, indicating that the score is an index of general similarity. When the scores of some historical typhoons are equally matched, the model first evaluates the dynamic sectors, and then weighs the static sectors, which reaffirms that RDP does matter. If the ranks still make no difference, the model keeps comparing by the order of the disparity of months, and the gap between the present year and the year of that historical typhoon. At that point, the similarity comparison result should be evident and reasonable.

## 4. Implementation

The research is implemented in the form of a web application, composed of a front-end information display panel and a back-end server developed using Flask, a Python library. The information display panel, which is the interface interacting with users, sends requests to the server, which is constructed based on Flask; the server starts an instant historical typhoon data renew, goes through procedures, including clean-up, track matching, etc., and returns formatted data and similarity rankings. Through this user-friendly interface, the concept of typhoon tracks matching in this research can be readily understood.

### 4.1. Data Source

**Figure 4:**
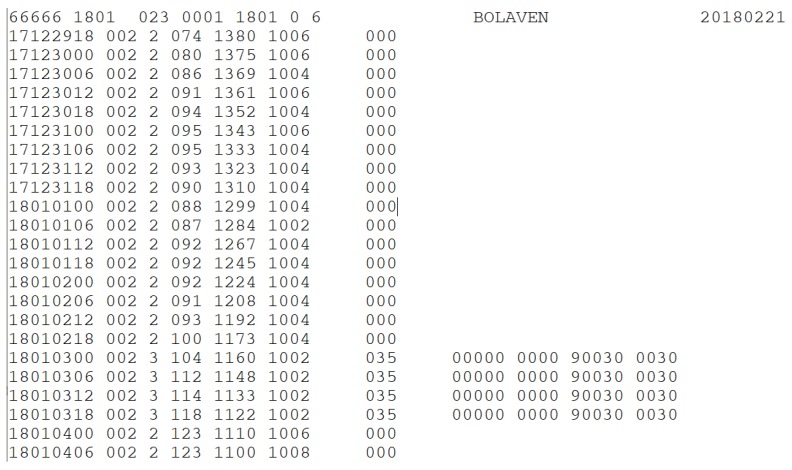
A sample of the Japan Meteorological Agency (JMA) best track data.

For TCs in the WNP, the historical best track data provided by the Japan Meteorological Agency (JMA), the regional specialized meteorological center of the WNP and the South China Sea, are the most used [17]. Tropical cyclone data in the jurisdiction of the JMA from 1951 to the present are collected from its open-access application programming interface (API). Each typhoon dataset has one header line and several data lines. As shown in Figure 4, each header line is composed of the international number and name of a typhoon, while the following data lines are the point data recorded every six hours, including its latitude, longitude, wind speed, etc. As Figure 4 shows, each line of data has a unique meaning in different positions. After line-by-line procedures, string processing and organization into a JSON (JavaScript object notation) format, the data are cleaned-up and well prepared for subsequent processing. As a result, the tropical cyclone data of the WNP for the past 70 years totaled approximately 1700 typhoons and contained approximately 65,000 coordinates.

Besides historical typhoons, present typhoon track forecasts are also required to analyze present typhoons at the information display stage. Several meteorological institutions provide forecasts of present typhoons in the WNP. In this study, we retrieved real-time forecasts from the Integrated Multi-Agency Tropical Cyclone Forecast, which provides comprehensive information about current typhoon forecasts from several meteorological institutions all over the world [18].

### 4.2. Information Display Panel

**Figure 5:**
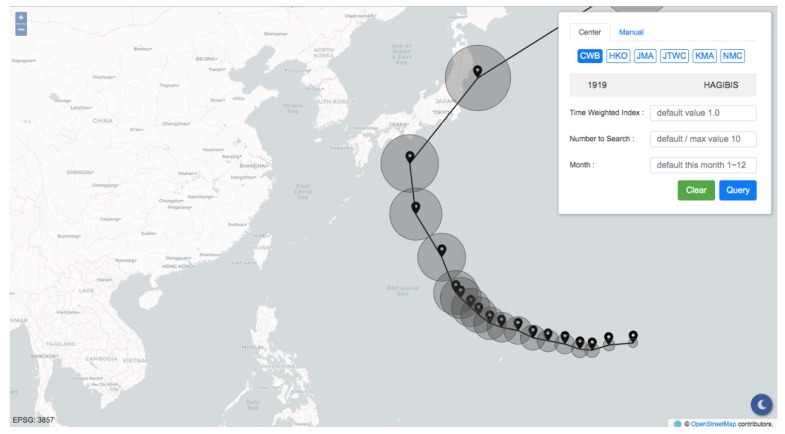
The information display panel.

The information display panel was developed using the HyperText Markup Language (HTML), Cascading Style Sheets (CSS), and ECMAScript (JavaScript). To deliver the primary objective of this research, the interface shown in Figure 5 contains only two components: a map built based on OpenLayers libraries and a control panel providing two modes for inputting track points.

The first mode is “center auto-import”, offering real-time feeds and forecasts of current typhoons from several meteorology institutions collected from the Integrated Multi-Agency Tropical Cyclone Forecast. The panel is capable of merging predictions from different meteorological institutions. When querying using the forecast track points retrieved from these institutions, there is a higher possibility that the query result matches the condition of the case, thus yielding greater accuracy.

The second mode is “manual input.” Users complete comparisons between any desired tracks and historical typhoon tracks. Track specification can be done by either keying in latitudes and longitudes or simply clicking on the map. The radii of valid regions can be determined by a tunable slide bar. At that point, essential parameters for the similarity comparison are ready, and those query conditions can be sent to the server by finally clicking on the button “query.” Hence, the query is accomplished intuitively and rapidly. As the server finishes the computation, the panel presents the result containing the basic information of the similar typhoons, including names, historical track points, and rankings. After being rendered, users can acquire historical typhoon tracks and rankings through the map and the table inside the panel. Furthermore, the typhoons listed in this table are attached with links to the corresponding information in the Taiwan Typhoon Database and can be selected independently.

**Figure 6:**
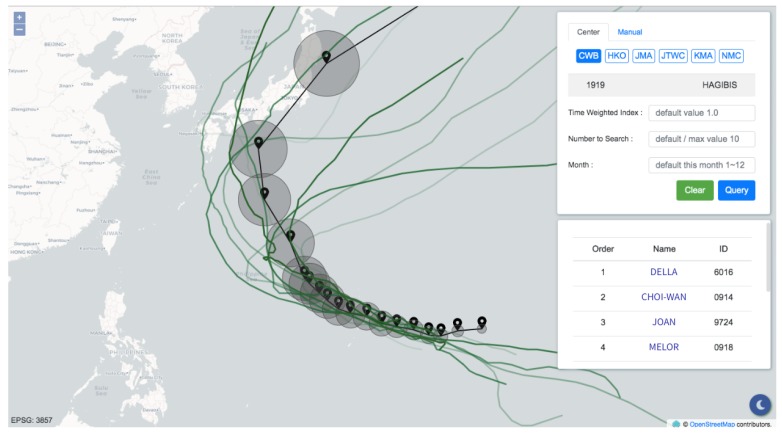
The query result using Typhoon Hagibis (2019).

The query result is illustrated in Figure 6. Through the two modes that the panel provides, users can not only comprehend the track similarities but also make a connection with what is going on. With the support of the panel, the application makes it convenient for decision makers to review historical typhoons quickly, which is a rapid way to grasp the current situation through the experience of past events. The damages caused by typhoons can thus be forecast, and precautions such as evacuations and pump pre-allocations can be executed on time.

## 5. Discussion

### 5.1. Effect of Time Weighting

In this section, we discuss the effect of setting different values for the time weighting. As described in Section 3.2, the dynamic sector of the model constructs a gRDW that ensures the dominance of recentness. To discuss the effect of setting different values of time weighting, this research relaxed RDP by allowing users to set the preferred time weighting. We divided the real number into seven special weight groups that give different interpretations of the time weighting. The most critical weight group is when *w* equals gRDW, which refers to the value determined by the number of user-specific track points and is the minimum value that ensures RDP. The remaining six groups are:

1. Over gRDW: w∈(gRDW,∞);
2. Positive related time weighting: w∈(0,gRDW);
3. Static sector: w=0, only presenting the static sector’s score;
4. Negative related time weighting: w∈(−1,0);
5. Repulsion force weighting: w=−1;
6. Reverse similarity: w∈(−∞,−1).

Since the six groups above are obtained by relaxing the presumption of RDP, setting time weightings with the six groups may not provide proper search results. The actual usage of non-RDP time weighting is not yet clarified and remains a future objective. However, in this research, we discuss the behavior of search results with time weightings of different groups, and the discussion provides insight for potential usage.

#### 5.1.1. Over gRDW

The over gRDW weight group refers to all the numbers greater than gRDW. For any time weightings set in this range, the outcome of the similarity model remains identical to the outcome’s time weighting set as gRDW. Thus, the time weightings in this group merely exaggerate RDP but generate the same result.

#### 5.1.2. Positive Relative Time Weighting

The positive relative time weighting group refers to the positive numbers that lie in the range of zero and gRDW. The positive relation highlights that for any time weighting set in this group, the recentness is proportionally increased; however, some of the cases that the recentness might be defeated by the static sector are erroneously included. The closer values are to gRDW, the more influential the recentness. The positive relative time weighting provides a trade off between the dynamic sector and the static sector.

#### 5.1.3. Static Sector

The static sector weight group refers to when the time weighting is set as zero, which eliminates the dynamic sector. Hence, the similarity comparison is solely determined by the static sector, which presents the pure summation of the user-specific track points passing through the valid regions of the historical typhoon track.

#### 5.1.4. Negative Relative Time Weighting

The negative relative time weighting group refers to negative time weightings that lie in the range of −1 and 0. The negative relation highlights that for any time weighting in this group, the recentness is proportionally decreased, making older typhoons play a more important role than recent typhoons. The closer to the −1 the time weighting is, the more influential the past is. However, the negative relative time weighting provides a limited interpretation of the comparison and prediction. The discussion merely points out the role that time weighting plays in this group.

#### 5.1.5. Repulsion Force Weight

The repulsion force weight group refers to when the time weight equals −1. This case is singled out for the reason that it generates the outcome resembling the graph of magnetic repulsion forces. When the time weight (*w*) is set as −1, the model reduces to:

(6) $$\sum_{k=1}^{M}\left(1+kw\right)=\sum_{k=1}^{M}\left(1-k\right)\leq 0.$$

Mathematically, the similarity score must be less than zero. Hence, the model must pick the historical typhoons that are scored as zero. Those historical typhoons whose scores are zero can be broken into two types:

1. Pass through none of the given points: $\sum_{k=1}^{M}\left(1-k\right)=0-0=0$;
2. Pass through the first given point: $\sum_{k=1}^{M}\left(1-k\right)=1-1=0$.

**Figure 7:**
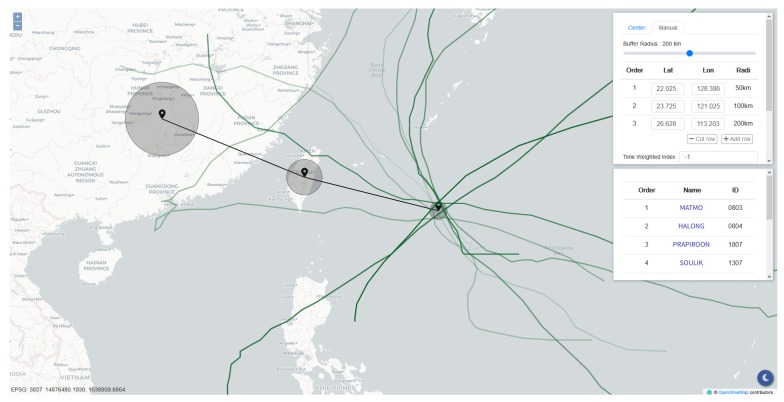
An example of the case of repulsion force weighting.

After sorting by the comparison order discussed in Section 3.4, the model prefers the latter more than the former, which would present results similar to the outcome shown by Figure 7. The graph resembles a magnetic field where the first given user input point acts as one of the magnetic poles while the other points act as the other poles. Consequently, the time weighting group for weights equals to –1 is named the repulsion force weight group.

#### 5.1.6. Reverse Similarity

The reverse similarity weighting group refers to the time weightings that are set as less than −1, which would make the model perfectly remove the effect of the user-specific track points. In other words, the model simply eliminates all the historical typhoons that pass through the user-specific track points. Hence, the model would simply skip the above three steps in the comparison order discussed in Section 3.4 and solely presents the historical typhoons that are scored as zero discussed in Section 5.1.5. Evidently, this result is contrary to this studies’ research goals.

### 5.2. Contributions

Several major advantages of this research over the preliminary work are evident.

First, this research discussed the details of the similarity model, developed a strategy to reduce computational requirements, developed a new information display panel featuring auto-importing typhoon forecast track data, and integrated the track similarity comparison technique and the typhoon database.

Next, in the similarity model, we not only considered the geometric similarity but also introduced the time weightings as a means of controlling the importance of track point time. We proposed the use of RDP regarding the effect of the number of track points. Furthermore, the effect of varying time weightings potentially provides a means for decision makers to modify the time weighting for different purposes.

Finally, we developed a new information display panel enabling the user to import forecast track data by both the manual-input method and the center auto-import method. The decision makers no longer need to specify the forecast track by hand; the new panel eliminates the possibility of making mistakes in the exact locations of the track points. In addition, we integrated several forecasters, providing various choices for the decision makers.

### 5.3. Limitations

There are several limitations of this research, including the lack of model performance validation, the potential effect of forecast error, and the ignorance of other important factors.

The validation of a model result with observations is currently not practicable because the factors for calculating the similarity of damages caused by multiple typhoon events remain unknown. Also, in the current design, the key parameters are decided by the decision makers in the implementation. To properly validate the model performance, the evaluation factors should be found, and the method of the decision maker setting up the key parameters should be discussed. In future work, more information should be collected from the application in real decision making cases to set up a reasonable validation process.

Since the historical typhoon search engine of this research relies on user-specific track points, the risk of error due to the forecast data cannot be eliminated. When there is a noticeable drop from the forecast and the reality, the search result derived from the forecast data may not apply well due to the existence of forecast error. To reduce the potential effect on relying on a single forecast, the search engine features auto-import forecast data from six different meteorology institutions. The decision makers can choose the preferred data source to utilize. By diversifying forecast data sources, the search engine can reduce the interference of underlying forecast error.

The search algorithm only considers track points as input. However, it is insufficient for decision support to leave other factors since the track is not the only factor that affects the damage. By taking other factors such as the intensity of typhoons into consideration, the search engine may be capable of getting a better search result that meets up to the current scenario. The ignorance of other factors is one of the limitations that need to be solved in future works.

## 6. Conclusions

This research improved on the preliminary work done on the similarity model and developed a new information display panel featuring auto-importing typhoon forecast track data. The similarity model provides an algorithm that compares all the historical typhoons tracks to the user-specific tracks. Compared with the historical typhoon tracks, when a typhoon is in its developing state, the decision makers can input the forecast track points and discover the historical typhoons that are observed to have the most similar characteristics to the upcoming typhoon. In this study, we introduced the recentness dominance principle (RDP), stating that recent data is always superior to older historical records. Following RDP, the similarity model computes the time weighting according to the number of user-specific points. Furthermore, the role of time weighting is discussed, providing direction for setting up the parameters for different purposes. The information display panel provides a concise and convenient interface for decision makers to quickly review historical typhoons, which is a rapid means to grasp the current situation through the experience of past events. 

## Figures and Tables

**Table 1:** All possible combinations for the case of M=5.

Static Sector	0	1	2	3	4	5
Possible combinations	0+0w	1+5w 1+4w 1+3w 1+2w 1+1w	2+9w 2+8w 2+7w 2+6w 2+5w 2+4w 2+3w	3+12w 3+11w 3+10w 3+9w 3+8w 3+7w 3+6w	4+14w 4+13w 4+12w 4+11w 4+10w	5+15w

**Table 2:** The indeterministic score pairs in the case of M = 5 and the calculation process of individual recentness dominance time weighting (iRDW).

$s_{1}+d_{1}w$	$s_{2}+d_{2}w$	$s_{1}+d_{1}w=s_{2}+d_{2}w$	iRDW
1+5w	2+4w 2+3w	1+5w=2+4w 1+5w=2+3w	w=1 $w=\frac{1}{2}$
1+4w	2+3w	1+4w=2+3w	w=1
2+9w	3+8w 3+7w 3+6w	2+9w=3+8w 2+9w=3+7w 2+9w=3+6w	w=1 $w=\frac{1}{2}$ $w=\frac{1}{3}$
2+8w	3+7w 3+6w	2+8w=3+7w 2+8w=3+6w	w=1 $w=\frac{1}{2}$
2+7w	3+6w	2+7w=3+6w	w=1
3+12w	4+11w 4+10w	3+12w=4+11w 3+12w=4+10w	w=1 $w=\frac{1}{2}$
3+11w	4+10w	3+11w=4+10w	w=1

**Table 3:** All possible combinations for the case of M=6.

Static Sector	0	1	2	3	4	5	6
Possible combinations	0+0w	1+6w 1+5w 1+4w 1+3w 1+2w 1+1w	2+11w 2+10w 2+9w 2+8w 2+7w 2+6w 2+5w 2+4w 2+3w	3+15w 3+14w 3+13w 3+12w 3+11w 3+10w 3+9w 3+8w 3+7w 3+6w	4+18w 4+17w 4+16w 4+15w 4+14w 4+13w 4+12w 4+11w 4+10w	5+20w 5+19w 5+18w 5+17w 5+16w 5+15w	6+21w

## Abbreviations

The following abbreviations are used in this manuscript:

CWB	Central Weather Bureau
gRDW	global recentness dominance time weighting
iRDW	individual recentness dominance time weighting
JMA	Japan Meteorological Agency
NII	National Institute of Informatics
RDP	recentness dominance principle
TC	tropical cyclone
WNP	western North Pacific
